# Multimodal integration of coagulation biomarkers and 3D Doppler ultrasound for diagnosis of placenta accreta spectrum in pernicious placenta previa: A prospective cohort study

**DOI:** 10.1097/MD.0000000000048810

**Published:** 2026-05-15

**Authors:** Man Gao, Yanan Yang, Liman Fu, Yanyan Peng, Yu Liang, Yingfeng Liu

**Affiliations:** a Department of Ultrasound, The Fourth Hospital of Shijiazhuang, Shijiazhuang, Hebei, China.

**Keywords:** D-dimer, diagnostic model, Doppler ultrasound, fibrinogen, pernicious placenta previa, placenta accreta spectrum, vascularization index

## Abstract

Pernicious placenta previa (PPP) complicated by placenta accreta spectrum (PAS) is associated with severe maternal morbidity and remains challenging to diagnose accurately before delivery. To evaluate the diagnostic performance of combining coagulation biomarkers (D-dimer [D-D], fibrinogen [FIB]) with three-dimensional Doppler ultrasound vascular indices for detecting PAS in PPP. In this prospective cohort study, 100 pregnant women with PPP were enrolled (PAS group: n = 66; control group: n = 34). D-D and FIB levels were measured in the second and third trimesters, alongside ultrasound-derived vascular indices, including vascularization index (VI), flow index (FI), and vascular flow index (VFI). Diagnostic performance was assessed using receiver operating characteristic (ROC) analysis, with intraoperative findings and/or histopathological confirmation as the reference standard. Patients with PAS exhibited significantly higher intraoperative blood loss, preterm birth rates, and transfusion requirements compared with controls (all *P* < .05). In the second trimester, both D-D and FIB levels were elevated in PAS (*P* < .05). In the third trimester, D-D levels further increased, whereas FIB levels decreased significantly (*P* < .05). Ultrasound vascular indices (VI, FI, and VFI) were consistently higher in the PAS group (*P* < .05). The combined model integrating D-D, FIB, and VI/FI/VFI demonstrated improved diagnostic performance, particularly in the third trimester, achieving an area under the curve of 0.952 with a sensitivity of 90.91% and specificity of 91.18%. Subgroup analysis showed that D-D levels increased and FIB levels decreased with increasing PAS severity, whereas ultrasound vascular indices did not differ significantly among PAS subtypes. The integration of coagulation biomarkers with Doppler-derived vascular indices significantly improves diagnostic accuracy for PAS in PPP. This multimodal approach provides a clinically useful tool for preoperative risk stratification and surgical planning.

## 1. Introduction

Pernicious placenta previa (PPP) complicated by placenta accreta spectrum (PAS) is associated with a high risk of antepartum hemorrhage, massive transfusion, and adverse perinatal outcomes, thereby posing a significant threat to maternal and fetal health.^[1]^ With the global rise in cesarean delivery rates, the incidence of PAS has increased markedly in recent decades, making it a growing challenge in modern obstetric practice.^[2]^ Accurate prenatal assessment of the depth and extent of placental invasion is therefore critical for optimizing surgical planning, preserving fertility, and improving long-term maternal outcomes.^[3]^

Currently, imaging modalities, particularly transvaginal ultrasound (TVUS), remain the cornerstone for evaluating PAS due to their noninvasive nature, real-time capability, and wide clinical availability.^[4]^ Advanced three-dimensional power Doppler techniques enable quantitative assessment of placental vascularization through parameters such as vascularization index (VI), flow index (FI), and vascular flow index (VFI), which reflect abnormal angiogenesis associated with invasive placentation.^[5]^ However, the diagnostic performance of ultrasound alone remains limited by operator dependency, fetal position, and variability in imaging interpretation, resulting in suboptimal sensitivity in certain clinical settings.^[6]^

Emerging evidence suggests that PAS is not only a structural disorder but also involves profound alterations in coagulation and fibrinolytic pathways. Disruption of abnormal placental vasculature may lead to microthrombosis and secondary hyperfibrinolysis, resulting in elevated levels of D-dimer (D-D), a marker of fibrin degradation.^[7]^ Concurrently, excessive consumption of coagulation factors at the uteroplacental interface, along with plasmin-mediated degradation, may lead to decreased or fluctuating fibrinogen (FIB) levels.^[8]^ These changes reflect a dynamic imbalance between hypercoagulation and hypocoagulation, providing a potential biochemical window into disease severity and bleeding risk.

Despite these insights, most previous studies have evaluated D-D or FIB either independently or in combination with a single imaging parameter, limiting their clinical applicability.^[9,10]^ Importantly, the potential synergistic value of integrating coagulation-fibrinolysisbiomarkers with quantitative ultrasound vascular indices remains largely unexplored. A multimodal approach that captures both molecular-level alterations and hemodynamic changes may substantially improve diagnostic accuracy and preoperative risk stratification. Furthermore, heterogeneity in diagnostic criteria and imaging protocols across centers underscores the need for standardized, prospective investigations to validate such integrated models.

Therefore, this prospective cohort study aimed to develop and evaluate a multimodal diagnostic model combining D-D, FIB, and three-dimensional Doppler ultrasound parameters (VI, FI, VFI) for the diagnosis of PAS in patients with PPP. By integrating biochemical and hemodynamic indicators, we hypothesized that this approach would enhance diagnostic performance and provide a more robust and clinically actionable tool for guiding surgical decision-making and improving patient outcomes.

## 2. Materials and methods

### 2.1. Study design and population

This single-center prospective cohort study was conducted in the Department of Obstetrics at The Fourth Hospital of Shijiazhuang, between April 2023 and June 2025.

Sample size estimation was performed using PASS 15.0 software based on diagnostic test design. Assuming an expected area under the receiver operating characteristic curve (AUC) of 0.90 for the combined model, with a significance level of α = 0.05, power of 90% (β = 0.10), and a case-to-control ratio of 2:1, the minimum required sample size was calculated as 87 participants. Accounting for a potential 10% dropout rate, a total of 100 patients were ultimately included.

### 2.2. Inclusion and exclusion criteria

Eligible participants met the following inclusion criteria:

(1) History of cesarean section (≥6 months prior to the current pregnancy);
(2) Diagnosis of placenta previa confirmed by transvaginal three-dimensional ultrasound, defined as placental tissue covering or reaching the internal cervical os;
(3) Availability of complete coagulation biomarker data (D-D and FIB) and ultrasound vascular parameters (VI, FI, VFI);
(4) Definitive diagnosis established by intraoperative findings and/or postoperative histopathology.

Exclusion criteria were:

(1) Multiple pregnancy or other placental disorders (e.g., placental abruption, placental tumors);
(2) Uncontrolled preexisting systemic diseases, including diabetes mellitus, hypertension, or autoimmune disorders;
(3) Inadequate ultrasound image quality preventing reliable assessment of placental implantation;
(4) Refusal to participate or loss to follow-up.

### 2.3. Reference standard and group classification

The reference standard for PAS was based on intraoperative findings and/or histopathological confirmation following cesarean delivery. PAS was defined according to the depth of placental villous invasion into the uterine wall.

Participants were categorized into 2 groups:

- PAS group (n = 66): including placenta accreta, placenta increta, and placenta percreta subtypes;
- Control group (n = 34): patients with placenta previa without evidence of abnormal placental invasion.

Subgroup classification of PAS severity was further performed for exploratory analysis.

### 2.4. Ultrasound assessment

All ultrasound examinations were performed using a Voluson E10 system (GE Healthcare, The Fourth Hospital of Shijiazhuang) equipped with transvaginal three-dimensional power Doppler capability. Scans were conducted by 2 experienced sonographers (>10 years of experience), blinded to clinical and laboratory data, to minimize observer bias.

Standardized imaging protocols were applied, including assessment of placental location, thickness, loss of the retroplacental clear zone, presence of placental lacunae, and disruption of the bladder line.

Three-dimensional vascular indices were obtained from the placental implantation site, including: VI, FI, and VFI.

For each patient, measurements were performed in 3 consecutive volumes, and the mean values were used for analysis to improve reliability.

### 2.5. Laboratory Measurements

Fasting venous blood samples were collected at 2 time points: second trimester (13–28 weeks of gestation) and third trimester (>28 weeks of gestation).

Plasma D-D and FIB levels were measured using an automated coagulation analyzer (Mindray C2000-A, China) according to the manufacturer’s protocols. All assays were performed in the hospital’s central laboratory under standardized conditions.

### 2.6. Clinical outcomes and study variables

Clinical outcomes included intraoperative blood loss, preterm birth rate, blood transfusion volume, and neonatal Apgar scores at 1 and 5 minutes.

Primary study variables comprised D-D, FIB, and ultrasound vascular indices (VI, FI, VFI). The primary endpoint was the diagnostic performance of these parameters, individually and in combination, for identifying PAS.

This study was conducted and reported in accordance with the STROBE (Strengthening the Reporting of Observational Studies in Epidemiology) guidelines.

### 2.7. Statistical analysis

Statistical analyses were performed using SPSS version 32.0 (IBM Corp., Armonk). Continuous variables were expressed as mean ± standard deviation and compared between groups using independent-samples *t* tests after assessment of normality. Categorical variables were presented as frequencies and percentages and compared using the chi-square test.

Diagnostic performance was evaluated using receiver operating characteristic (ROC) curve analysis, with calculation of the area under the curve (AUC), sensitivity, and specificity. Optimal cutoff values were determined based on the maximum Youden index (sensitivity + specificity − 1).

Multivariable logistic regression analysis was performed to assess the combined diagnostic value of D-D, FIB, and ultrasound vascular indices, including VI, FI, and VFI. The combined model was constructed as follows:


$$\begin{array}{l} Logit(P)=\beta_{0}+\beta_{1}(D-D)+\beta_{2}(FIB)+\beta_{3}(VI)+\beta_{4}(FI)+\beta_{5}(VFI), \end{array}$$

where P represents the predicted probability of PAS. The diagnostic performance of the combined model was evaluated using ROC analysis.

A two-tailed *P* value < .05 was considered statistically significant.

### 2.8. Ethics approval and consent to participate

This study was approved by the Institutional Ethics Committee of the Fourth Hospital of Shijiazhuang (Approval No. 20230143) and conducted in accordance with the principles of the Declaration of Helsinki. Written informed consent was obtained from all participants prior to inclusion in the study.

## 3. Results

### 3.1. Baseline characteristics

A total of 100 pregnant women with PPP were included, comprising 66 patients in the PAS group and 34 in the control group. Baseline characteristics, including maternal age, gravidity, parity, gestational age at enrollment, prior history of PPP, and placental location, were comparable between the 2 groups (all *P* > .05), indicating good baseline balance (Table 1).

**Table 1:** Baseline characteristics of the study population.

Variable	Control (n = 34)	PAS (n = 66)	*P*-value	SMD
Age (yr)	32.29 ± 4.26	32.77 ± 3.90	.575	0.120
Gravidity (n)	3.15 ± 0.82	3.00 ± 0.82	.399	0.183
Parity, n (%)				
1	14 (41.18)	22 (33.33)	.439	0.242
>1	20 (58.82)	44 (66.67)		
Gestational age (wk)	21.44 ± 2.27	20.98 ± 1.79	.273	0.235
History of PPP, n (%)				
Yes	5 (14.71)	13 (19.70)	.538	0.173
No	29 (85.29)	53 (80.30)		
Placental location, n (%)				
Anterior wall	10 (29.41)	22 (33.33)	.911	0.154
Posterior wall	17 (50.00)	32 (48.48)		
Lateral wall	7 (20.59)	12 (18.18)		

Data are presented as mean ± standard deviation or n (%).

*P*-values were calculated using independent-samples t test or chi-square test as appropriate.

PAS = placenta accreta spectrum, PPP = pernicious placenta previa, SMD = standardized mean difference.

### 3.2. Clinical outcomes

Compared with the control group, patients with PAS experienced significantly greater intraoperative blood loss (924.47 ± 188.30 vs 799.42 ± 153.61 mL, *P* = .001), higher rates of preterm birth (36.36% vs 14.71%, *P* = .024), and increased blood transfusion volumes (673.42 ± 120.21 vs 600.33 ± 107.17 mL, *P* = .004).

No significant differences were observed in neonatal Apgar scores at 1 minute (7.61 ± 1.14 vs 7.82 ± 1.33, *P* = .411) or 5 minutes (8.30 ± 0.96 vs 8.61 ± 1.00, *P* = .147) between the 2 groups (Table 2).

**Table 2:** Clinical outcomes.

Outcome	Control (n = 34)	PAS (n = 66)	*P*-value
Intraoperative blood loss (mL)	799.42 ± 153.61	924.47 ± 188.30	.001
Preterm birth, n (%)	5 (14.71)	24 (36.36)	.024
Blood transfusion volume (mL)	600.33 ± 107.17	673.42 ± 120.21	.004
Apgar score (1 min)	7.82 ± 1.33	7.61 ± 1.14	.411
Apgar score (5 min)	8.61 ± 1.00	8.30 ± 0.96	0.147

Data are presented as mean ± standard deviation or n (%).

*P*-values were calculated using independent-samples *t* test or chi-square test as appropriate.

PAS = placenta accreta spectrum.

### 3.3. Diagnostic performance of individual and combined biomarkers

Table 3 shows that individual biomarkers and ultrasound vascular indices demonstrated moderate to good ability to discriminate PAS, with AUC values ranging from approximately 0.739 to 0.902, indicating that no single parameter was sufficiently robust for standalone clinical diagnosis. Among the individual indices, third-trimester D-D and FIB showed improved performance compared with their second-trimester counterparts, while VFI exhibited the highest diagnostic value among the ultrasound parameters.

**Table 3:** Diagnostic performance of individual and combined markers for PAS in PPP patients.

Parameter	AUC	95% CI	Sensitivity (%)	Specificity (%)
Second trimester				
D-D	0.784	0.695–0.873	72.73	76.47
FIB	0.739	0.641–0.837	66.67	70.59
VI	0.809	0.725–0.893	75.76	79.41
FI	0.775	0.684–0.866	69.70	76.47
VFI	0.826	0.747–0.905	77.27	82.35
Combined model	0.888	0.81–0.95	81.82	87.88
Third trimester				
D-D	0.839	0.761–0.917	78.79	79.41
FIB	0.792	0.711–0.873	72.73	76.47
VI	0.874	0.801–0.947	83.33	85.29
FI	0.828	0.749–0.907	77.27	79.41
VFI	0.902	0.842–0.962	86.36	88.24
Combined model	0.952	0.91–0.99	90.91	91.18

Cutoff values were determined using the maximum Youden index.

Combined model derived from multivariable logistic regression.

AUC = area under the curve, CI = confidence interval, D-D = D-dimer, FI = flow index, FIB = fibrinogen, PAS = placenta accreta spectrum, PPP = pernicious placenta previa, VFI = vascular flow index, VI = vascularization index.

In contrast, combining coagulation biomarkers with Doppler vascular indices markedly improved diagnostic performance. The second-trimester combined model achieved good discrimination (AUC = 0.888), whereas the third-trimester combined model demonstrated excellent accuracy, with an AUC of 0.952, sensitivity of 90.91%, specificity of 91.18%, and the highest Youden index (0.821). These findings indicate that multimodal assessment substantially outperforms individual markers and that third-trimester combined evaluation provides the greatest clinical utility for identifying PAS.

Multivariable logistic regression analysis demonstrated that D-D, FIB, and ultrasound vascular indices (VI and VFI) were independent predictors of PAS (Table 4). Increased D-D levels and higher vascular indices were significantly associated with an increased risk of PAS, whereas lower FIB levels were associated with a higher likelihood of disease. FI showed a positive association with PAS but did not reach statistical significance. These findings support the complementary diagnostic roles of coagulation biomarkers and placental vascularization parameters (Table 4).

**Table 4:** Multivariable logistic regression analysis for prediction of PAS.

Variable	β coefficient	Standard error (SE)	Odds ratio (OR)	95% CI for OR	*P*-value
D-dimer (D-D)	0.87	0.26	2.39	1.43–3.99	.001
Fibrinogen (FIB)	−0.68	0.24	0.51	0.32–0.82	.005
Vascularization index (VI)	0.74	0.21	2.10	1.39–3.16	<.001
Flow index (FI)	0.31	0.18	1.36	0.96–1.94	.081
Vascular flow index (VFI)	0.95	0.29	2.58	1.47–4.54	.001
Intercept (β_0_)	−5.28	–	–	–	–

### 3.4. Changes in coagulation biomarkers and ultrasound parameters

Significant differences in coagulation biomarkers and ultrasound vascular indices were observed between groups.

In the second trimester, both D-D and FIB levels were significantly higher in the PAS group compared to controls (*P* < .05). In the third trimester, D-D levels remained elevated and further increased in PAS patients, whereas FIB levels were significantly lower than those in the control group (*P* < .05).

Within the PAS group, D-D levels increased significantly from the second to the third trimester, while FIB levels showed a significant decline (*P* < .05), indicating dynamic changes in coagulation status over gestation.

**Figure 1.**
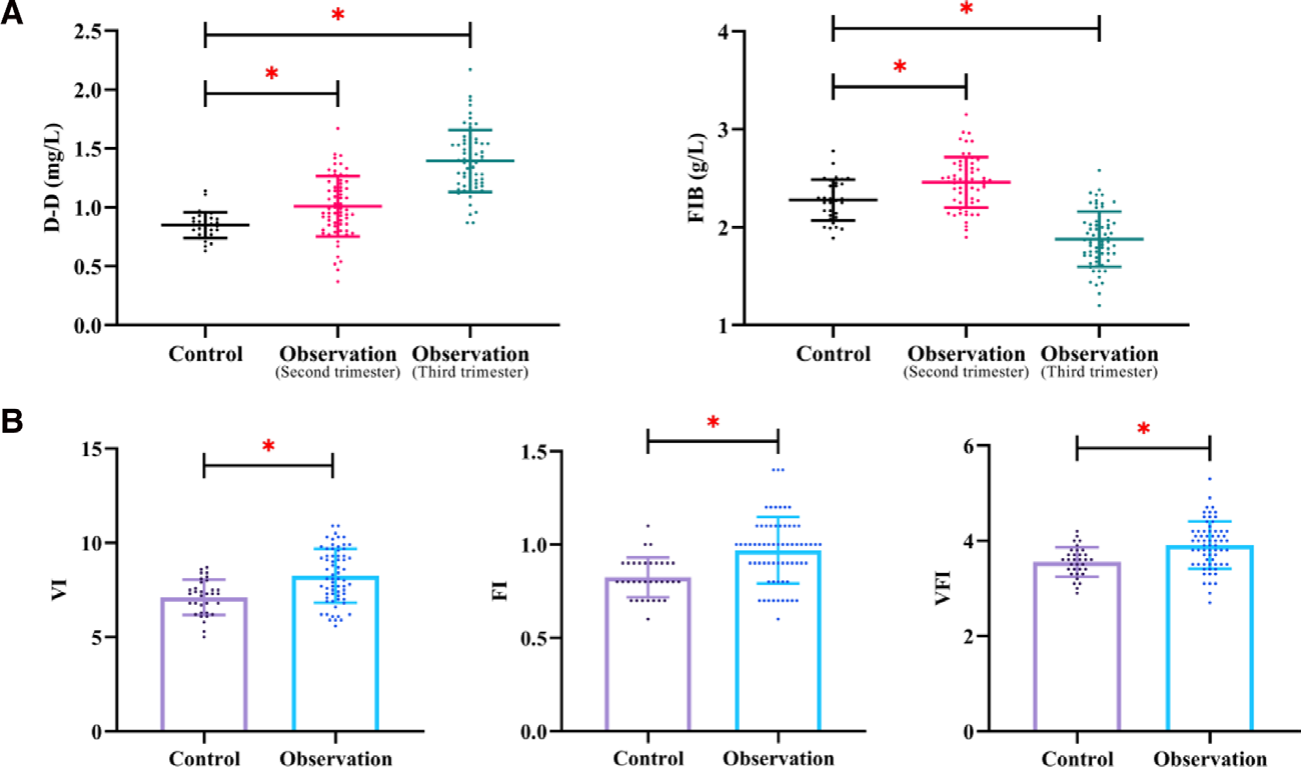
Comparison of coagulation biomarkers and ultrasound vascular indices between PAS and control groups. (A) Scatter plots showing D-dimer (D-D) and fibrinogen (FIB) levels in control subjects and PAS patients during the second and third trimesters. D-D levels were significantly elevated in PAS and increased further in the third trimester, whereas FIB levels showed a decreasing trend in late pregnancy. (B) Comparison of ultrasound-derived vascular indices, including vascularization index (VI), flow index (FI), and vascular flow index (VFI), between control and PAS groups. All vascular parameters were significantly higher in PAS patients, indicating increased placental vascularization. PAS = placenta accreta spectrum, FIB = fibrinogen.

In addition, ultrasound-derived vascular indices, including VI, FI, and VFI, were significantly higher in the PAS group compared with controls (all *P* < .05), reflecting increased placental vascularization (Fig. 1).

### 3.5. Subgroup analysis by PAS severity

Subgroup analysis revealed no significant differences in ultrasound vascular indices (VI, FI, VFI) among placenta accreta, placenta increta, and placenta percreta subtypes (*P* > .05).

Similarly, no significant differences in D-D or FIB levels were observed among subgroups in the second trimester (*P* > .05). However, in the third trimester, both biomarkers showed significant variation across PAS severity (*P* < .05).

**Figure 2.**
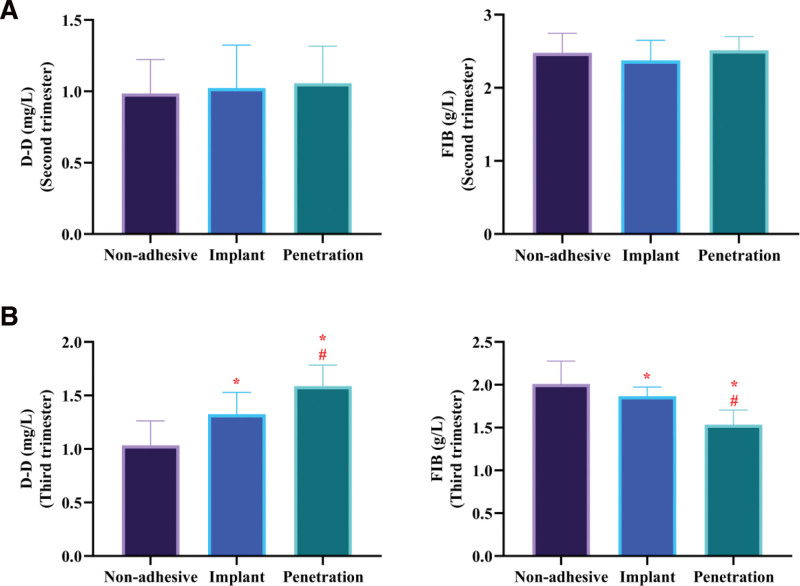
Subgroup analysis of coagulation biomarkers across different PAS severities. (A) Comparison of D-dimer (D-D) and fibrinogen (FIB) levels among placenta accreta, placenta increta, and placenta percreta subtypes in the second trimester. No significant differences were observed among subgroups. (B) Comparison of D-D and FIB levels among PAS subtypes in the third trimester. D-D levels increased progressively with disease severity, whereas FIB levels showed a decreasing trend. Data are presented as mean ± standard deviation. *P < .05 compared with the placenta accreta group; ^#^*P* < .05 compared with the placenta increta group. PAS = placenta accreta spectrum.

Specifically, D-D levels were highest in penetrating PAS, followed by invasive and adherent subtypes, whereas FIB levels demonstrated the opposite trend, with the lowest values observed in penetrating PAS. These findings suggest that coagulation biomarkers are associated with disease severity, particularly in late pregnancy (Fig. 2).

### 3.6. Receiver operating characteristic analysis

ROC analysis demonstrated that individual parameters, including D-D, FIB, and ultrasound vascular indices (VI, FI, and VFI), provided moderate to good diagnostic performance for identifying PAS. Among these, ultrasound-derived VFI exhibited the highest diagnostic value, particularly in the third trimester.

**Figure 3.**
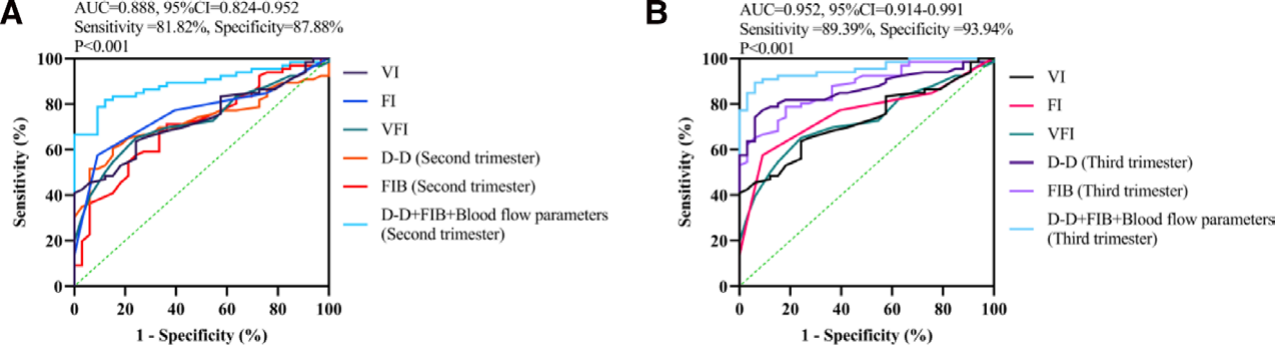
Receiver operating characteristic (ROC) curves of individual and combined diagnostic parameters for placenta accreta spectrum (PAS). (A) ROC curves for D-dimer (D-D), fibrinogen (FIB), and ultrasound vascular indices (VI, FI, VFI) in the second trimester. The combined model demonstrated good diagnostic performance (AUC = 0.888, 95% CI: 0.824–0.952), with a sensitivity of 81.82% and specificity of 87.88%. (B) ROC curves for D-D, FIB, and ultrasound vascular indices in the third trimester. The combined model showed excellent diagnostic performance (AUC = 0.952, 95% CI: 0.914–0.991), with a sensitivity of 90.91% and specificity of 91.18%. The diagonal dashed line represents the reference line (AUC = 0.5). The combined model integrates D-D, FIB, and ultrasound blood flow parameters. CI = confidence interval, FI = flow index, VI = vascularization index.

In contrast, the combined diagnostic model integrating coagulation biomarkers with Doppler vascular indices significantly improved diagnostic accuracy. In the second trimester, the combined model achieved good discrimination, with an AUC of 0.888, sensitivity of 81.82%, and specificity of 87.88%. The combined model showed excellent diagnostic performance (AUC = 0.952, 95% CI: 0.914–0.991), with a sensitivity of 90.91% and specificity of 91.18% (Fig. 3).

## 4. Discussion

This study demonstrates that the integration of coagulation–fibrinolysis biomarkers with three-dimensional Doppler ultrasound vascular indices significantly improves the diagnostic performance for PAS in patients with PPP. In this prospective cohort, the combined model achieved excellent discrimination, with an AUC of 0.952, outperforming individual biomarkers and ultrasound parameters. These findings further support the concept that PAS is a multifactorial disorder involving both biochemical and hemodynamic alterations, and that a multimodal diagnostic approach can enhance preoperative identification and risk stratification.

Consistent with the clinical severity of PAS, patients in the PAS group exhibited significantly greater intraoperative blood loss, higher rates of preterm birth, and increased transfusion requirements. These observations are in line with previous studies demonstrating that abnormal placental invasion disrupts uterine architecture and predisposes to massive hemorrhage during delivery.^[11–13]^ The lack of significant differences in neonatal Apgar scores may reflect improvements in perioperative management, early diagnosis, and timely multidisciplinary intervention.

A key finding of this study is the dynamic alteration of coagulation biomarkers across gestation. D-D levels were significantly elevated in PAS patients in both the second and third trimesters, with a further increase observed in late pregnancy. In contrast, FIB levels demonstrated a biphasic pattern, with relative elevation in the second trimester followed by a significant decline in the third trimester. These findings highlight a progressive imbalance between coagulation activation and fibrinolysis during the development of PAS. Mechanistically, abnormal trophoblastic invasion into the myometrium leads to extensive vascular remodeling, endothelial disruption, and localized microthrombosis.^[14,15]^ Elevated D-D levels reflect increased fibrin turnover and secondary hyperfibrinolysis, whereas decreased FIB levels in advanced disease likely result from excessive consumption of coagulation factors and impaired compensatory synthesis.^[7,16]^ Notably, in the placenta percreta subgroup, D-D levels were highest and FIB levels were lowest, further supporting their association with disease severity and hemorrhagic risk.

Ultrasound-derived vascular indices (VI, FI, and VFI) were consistently higher in PAS patients, reflecting increased placental vascularization and perfusion. These findings are consistent with prior reports linking invasive placentation to aberrant angiogenesis and vascular hyperproliferation.^[17,18]^ However, no significant differences in these indices were observed among PAS subtypes, suggesting that Doppler vascular parameters may primarily capture the extent of placental vascularization rather than the depth of myometrial invasion.^[19,20]^ This limitation underscores the importance of complementary biomarkers to better characterize disease severity.

Importantly, individual diagnostic markers demonstrated only moderate to good performance, whereas the multimodal model achieved a substantial improvement in diagnostic accuracy. This enhancement likely arises from the complementary nature of the integrated parameters. Specifically, D-D and FIB reflect molecular and biochemical alterations in coagulation–fibrinolysis pathways, while VI, FI, and VFI provide quantitative assessment of placental vascular architecture and perfusion. By integrating these orthogonal dimensions, the model captures both functional and structural aspects of PAS, thereby reducing diagnostic uncertainty associated with single-modality approaches.

Another important observation is the superior performance of the combined model in the third trimester compared with the second trimester. This finding suggests that both coagulation imbalance and abnormal vascular proliferation become more pronounced as gestation progresses, enabling more accurate detection of advanced disease. Therefore, dynamic monitoring of these biomarkers particularly in late pregnancy may be critical for identifying high-risk patients and optimizing the timing and planning of delivery.

From a clinical perspective, this study provides a practical and quantifiable multimodal framework for the preoperative assessment of PAS in PPP. The integration of routinely available laboratory biomarkers with ultrasound vascular indices may facilitate earlier risk stratification, support individualized surgical planning, and potentially reduce maternal morbidity. In particular, identifying high-risk patients in the third trimester may inform decisions regarding delivery timing, surgical approach, and resource allocation, including the need for multidisciplinary teams and blood product preparation.^[1,3]^ Importantly, this study highlights the clinical feasibility of integrating routinely available laboratory biomarkers with advanced ultrasound techniques in real-world settings.

### 4.1. Study strengths

This study has several notable strengths. First, it adopts a prospective cohort design with clearly defined inclusion criteria and standardized data collection, which enhances the internal validity of the findings. Second, the study integrates both biochemical (D-D and FIB) and imaging-based (three-dimensional Doppler vascular indices) parameters, providing a comprehensive and clinically relevant multimodal diagnostic framework. Third, ultrasound assessments were performed by experienced operators using standardized protocols and blinded to laboratory results, minimizing measurement bias. Fourth, the use of objective reference standards based on intraoperative findings and/or histopathological confirmation strengthens diagnostic accuracy. Finally, the dynamic evaluation of biomarkers across the second and third trimesters provides novel insights into the temporal evolution of coagulation changes in PAS.

### 4.2. Limitations

Several limitations should be acknowledged. First, this was a single-center study with a relatively small sample size, particularly in the placenta percreta subgroup, which may limit generalizability and statistical power. Second, the combined diagnostic model was developed and evaluated within the same cohort, which may introduce optimism bias; external validation in independent populations is required. Third, although ultrasound assessments were performed by experienced operators, measurement variability related to fetal position, placental location, and acquisition settings cannot be completely excluded. Fourth, biomarker measurements were limited to the second and third trimesters, and early gestational changes were not assessed. Future multicenter studies with larger cohorts, standardized imaging protocols, and longitudinal biomarker evaluation are warranted. In addition, incorporation of emerging biomarkers and advanced analytical approaches, such as machine learning–based imaging analysis, may further improve diagnostic accuracy and reproducibility.

## 5. Conclusion

The integration of coagulation biomarkers (D-D and FIB) with three-dimensional Doppler ultrasound vascular indices (VI, FI, and VFI) significantly improves the diagnostic performance of PAS in patients with PPP. By combining biochemical and hemodynamic information, this multimodal approach provides a more comprehensive assessment of disease status and represents a practical tool for preoperative risk stratification and clinical decision-making. Notably, the diagnostic accuracy of the combined model was highest in the third trimester, suggesting that progressive alterations in coagulation–fibrinolysis balance and placental vascularization enhance detectability in late gestation. Therefore, dynamic monitoring of these parameters may facilitate more accurate identification of high-risk patients and support timely intervention. Future studies should focus on external validation of this model in larger, multicenter cohorts, as well as the integration of additional biomarkers and advanced analytical techniques to further improve predictive performance and enable more precise diagnosis and management of PAS.

## Author contributions

**Investigation:** Man Gao, Yanan Yang, Liman Fu, Yanyan Peng, Yu Liang, Yingfeng Liu.

**Methodology:** Man Gao, Yanan Yang, Liman Fu, Yanyan Peng, Yu Liang, Yingfeng Liu.

**Writing – review & editing:** Liman Fu.
